# Potential improvements of the cognition of piglets through a synbiotic supplementation from 1 to 28 days via the gut microbiota

**DOI:** 10.1038/s41598-021-03565-5

**Published:** 2021-12-16

**Authors:** Severine P. Parois, Susan D. Eicher, Stephen R. Lindemann, Jeremy N. Marchant

**Affiliations:** 1 PEGASE, Agrocampus Ouest, INRA, Saint-Gilles, France; 2 USDA-ARS, Livestock Behavior Research Unit, West Lafayette, IN USA; 3 Department of Food Science, Purdue University, West Lafayette, IN 47907 USA

**Keywords:** Microbiology, Zoology

## Abstract

The influence of feed supplements on behavior and memory has been recently studied in livestock. The objectives of the study were to evaluate the effects of a synbiotic on: an episodic-like (SOR: Spontaneous Object Recognition), a working (BARR: Fence barrier task), a long-term (TMAZE: Spatial T-maze task) memory test and on gut microbiota composition. Eighteen female piglets were supplemented from 1 to 28 days of age with a synbiotic (SYN), while 17 served as control (CTL). Feces were collected on days 16, 33 and 41 for 16S rRNA gene composition analyses. In the SOR, SYN piglets interacted more quickly with the novel object than CTL piglets. In the BARR, SYN piglets had shorter distances to finish the test in trial 3. In the TMAZE, SYN piglets were quicker to succeed on specific days and tended to try the new rewarded arm earlier during the reversal stage. Difference of microbiota composition between treatments was nonexistent on D16, a tendency on D33 and significant on D41. The synbiotic supplement may confer memory advantages in different cognitive tasks, regardless of the nature of the reward and the memory request. Difference in memory abilities can potentially be explained by differences in microbiota composition.

## Introduction

Although cognition in farm animals is a relatively recent field of study, there is a growing body of evidence to suggest that pigs possess complex physical and social cognitive abilities, which has implications for their housing, their husbandry, and their welfare^1^. There is also growing evidence of an interaction between brain and gut microbiota^2^ so that by possible neural, endocrine and immune pathways, the gut microbiota communicates with the central nervous system to affect brain function and the behavior, mood and cognition of the individual host^3^.

A piglet’s gastrointestinal tract is postulated to be devoid of microbiota at the time of birth^4^, but becomes rapidly populated during and after the birth process^5^. As the piglet ages, stable, dominant microbial populations arise for the first 3 weeks of life, but with increasing minor populations establishing and contributing to overall diversity^5^. Multiple superimposed stressors occur at weaning, which for commercial pigs occurs around 3–4 weeks of age, including maternal separation, an abrupt change in diet from milk to solid food, and mixing into groups with unfamiliar pen-mates^6^. These combined stressors often result in post-weaning diarrhea and compromised welfare post-weaning^7^, often attributed to *E. coli* proliferation in the gut^8^, and which has resulted in routine inclusion of antibiotics in the post-weaning diet.

As societal concerns about antimicrobial resistance have increased, there is a need to investigate alternatives to antibiotics, and interest in probiotics has surfaced. *Lactobacillus* species have been commonly used in this capacity, given their ability to encourage growth of a healthy microbiota and exclusion of enteric pathogens^9^. However, it is not known whether supplementation with lactobacilli may affect behavior and, especially, cognitive abilities. The aims of this study therefore were to determine whether supplementation with a synbiotic (combined pre- and probiotic) before and after weaning would affect piglet cognition and gut microbial populations.

## Materials and methods

The experiment was approved by the local ethical committee Purdue University Animal Care and Use Committee (authorization number #1602001367A003) and was carried out in accordance with relevant guidelines and regulations. All methods applied in the study were performed in accordance with the ARRIVE guidelines and regulations. Due to the administration of different supplementations according to the treatment group, the investigator was aware of the treatment diet of the animals. The microbiota composition analyses were done blind. The sample size estimation was based on the number of animals required for memory evaluation and behavioural part of the study using similar results obtained in pigs subjected to different treatments (α = 5%, power = 80%, SD = 0.2, δ = 0.2)^10,11^.

### Animals and housing

A total of 35 female crossbred Duroc × (Landrace × Yorkshire) piglets from 17 different litters (2 or 3 piglets per sow) were reared under the same conditions from birth to the end of the experiment 41 days later. Sows were housed in 0.6 m × 2.3 m long farrowing crates within 1.5 m × 2.7 m pens, with fully-slatted floors and solid pen partitions. The two/three female piglets were selected on the basis of health status and birth weight relative to average litter birth weight. Farm processing procedures (teeth clipping, iron injecting and tail docking) occurred at 2.4 ± 0.9 days of age (mean ± SD), and piglets were weaned at 18.1 ± 1.8 days of age. At weaning, 3 females were removed from the study and the 32 remaining females (16 per treatment) were divided into 4 nursery pens 120 × 140 cm (8 non-littermate piglets per pen and 2 pens per treatment), splitting the pair originating from the same litter to spread out any potential genetic effect. All pens had fully-slatted floors and contained one 5-hole dry self-feeder and a cup waterer to allow for ad libitum access to feed and water. Synbiotic treatment and control treatment pens were separated by empty pens to avoid potential cross contamination between animals from the different treatments, since piglets in adjoining pens could have some tactile contact through the gated pen partitions. The 4 pens were equally spread over the experimental room in order to avoid any confusion between the treatment applied and the ambiance in the room.

### Feeding management and synbiotic distribution

The day after birth, litter was assigned to one of the two following treatments: Synbiotic treatment (SYN) consisting of an oral supplement of 5 ml, given individually by syringes in chocolate milk (TruMoo Chocolate—Whole milk), containing probiotics (3 strains of *Lactobacillus* at 10^9^ CFU/piglet), prebiotics (fructo-oligosaccharide at 10 mg/day/piglet—Sigma Aldrich; St. Louis, MO), a dietary fiber (beta-glucan at 11 mg/day/piglet—Sigma Aldrich; St. Louis, MO) and vitamin C (at 10 mg/day/piglet); Control treatment (CTL) consisting of 5 ml of chocolate milk. The *Lactobacillus* strains were chosen from 12 *Lactobacillus* isolates from piglet jejunal contents, with incubation and isolation carried out in the USDA-ARS lab (10? Petrosus, 2016). Through the use of gel electrophoresis, identification, and measuring concentrations of various neurochemicals produced, 3 isolates were selected based on potential anxiolytic properties. The 3 isolates were identified as *Lactobacillus salivarius, Lactobacillus reuteri,* and *Lactobacillus reuteri*, respectively.

To dose the piglets with the respective treatment, piglets were held in an experimenter’s arms while another experimenter administrated the liquid in its mouth. The total duration of the procedure did not exceed 30 s. The synbiotic was given from birth to weaning 5 days/week (M, T, W, F, S) and every single day after weaning until 28 days of age. Until weaning, piglets were in stable social and external microbial environments, which may favor stability in the gut microbiota. At weaning, solid food was introduced and available ad libitum, together with water. The base diets were similar for both treatments and were formulated to meet nutritional requirements based on piglet BW. The previously mentioned social mixing, sow separation and dietary change are responsible for an intense stress for piglets and consequent disturbances of the microbiota composition. To attempt to mitigate this disturbance, supplementation with the synbiotic every single day was administered.

### Behavioral tests

Pigs were subjected to 3 distinct cognitive tests: (1) a spontaneous object recognition test (SOR) to evaluate episodic-like memory at 16 days of age; (2) a fence barrier task (BARR) to evaluate problem-solving skills at 20 days of age; (3) a spatial T-maze task (TMAZE) to evaluate spatial learning and memory from 33 to 41 days of age.

#### Habituation phase

In order to acclimate the piglets to isolation, piglets were subjected to periods of isolation from 12 to 14 days of age in 1 of the 2 empty arenas (L208 × W137 × H82cm) located in the adjacent room. The floor of both arenas was standardized using a black heavy-duty rubber mat. Because of possible bias due to differences in location within the room, each piglet was assigned to one of the arenas, in which it did all the relevant tests. The habituation to isolation was carried out by a single experimenter with two sessions per day, separated at least by a 45 min break during which piglets were taken back to their home pen, from 9:00 h to 19:00 h (random assignment to mornings or afternoons). Introduction of the piglets in the arena was always done at the same location of the arena, in the periphery of the arena on the long side. The process followed a progressive pattern: day 1–10 min in a pair then 5 min alone; day 2–5 min alone then 10 min alone; day 3–10 min alone twice. The variables checked were: the total distance travelled, the frequency and the percentage of time spent in periphery (within 25 cm of the walls), the number of jumps and the latency to jump for the first time.

#### Spontaneous object recognition test (SOR)

A spontaneous object recognition test was used to evaluate episodic-like memory in piglets at 16 days of age^10,12,13^. This test is a non-feed motivation test. It is based on the innate ability of pigs to be attracted and explore objects. Considering the innate preference of pigs for novelty, a preference to explore a novel object in comparison to a familiar one is considered as a proof of memorization. However, because of the limited opportunities for the piglets to perform exploration of objects in their basic home pen environment, an orange plastic stick (H28 × Ø4 cm) was attached to the back of the farrowing crate in the home pen about 24 h before the novel object recognition test to encourage them to learn to explore a novel object.

The test was carried out during the afternoon between 12:00 h to 20:00 h in the same arena that habituation occurred. The test consisted of two sessions. During the first 10-min session, the piglet was exposed to two similar objects (O1 and O2) attached to each of the side walls in the arena. Then, the piglet was returned to its home pen for a 50-min break. During the second 10-min session, the piglet was exposed to one familiar object (O3) and a completely new one (N1). All the handling of the tested piglet was done by the same familiar experimenter to avoid any supplemental stress. The two different objects used were of the exact same red color and silicone material, with two different shapes: a pasta spoon (L28 × W4 cm) and a spoon rest (L24 × W12 cm). They were attached 14 cm above the floor and 20 cm from the corner of the arena. Objects were washed with soap and water and dried between each trial to minimize odorous cues. The object type and the side of novelty were randomized between piglets. A semi-circular area was designated around the object on both sides (Ø100 cm, with the center matching the middle of the object). The test was videotaped and analyzed by a single trained observer blind to treatment using Ethovision XT 13^14^. The variables analyzed were as follows: the latencies, frequencies and total durations of interactions with both objects (DN1: duration with the novel object; DO3: duration with the old object), position in the designated area next to the novel objects, as well as a discrimination index (DI) = (DN1 − DO3)/(DN1 + DO3)^15^.

#### Fence barrier task (BARR)

A fence barrier task was used to evaluate problem-solving skills and short-term memory at 16 days of age, based on visual clues. This test was adapted from the glass barrier test from Friess et al.^16^. The glass obstacle was substituted by chicken wire fence because of concerns that light reflections from the glass could possibly scare the piglets or impair their visibility of the reward. The feed-motivation reward was substituted by a social-motivation reward using two companion piglets coming from the same litter. It is based on the gregarious instinct of pigs. The unique glass barrier was substituted by two fence barriers in order to make the task more complex. The fences had 25 cm holes on one side (opposite sides for the two fences) to enable the piglet to access the next section of the area.

**Figure 1:**
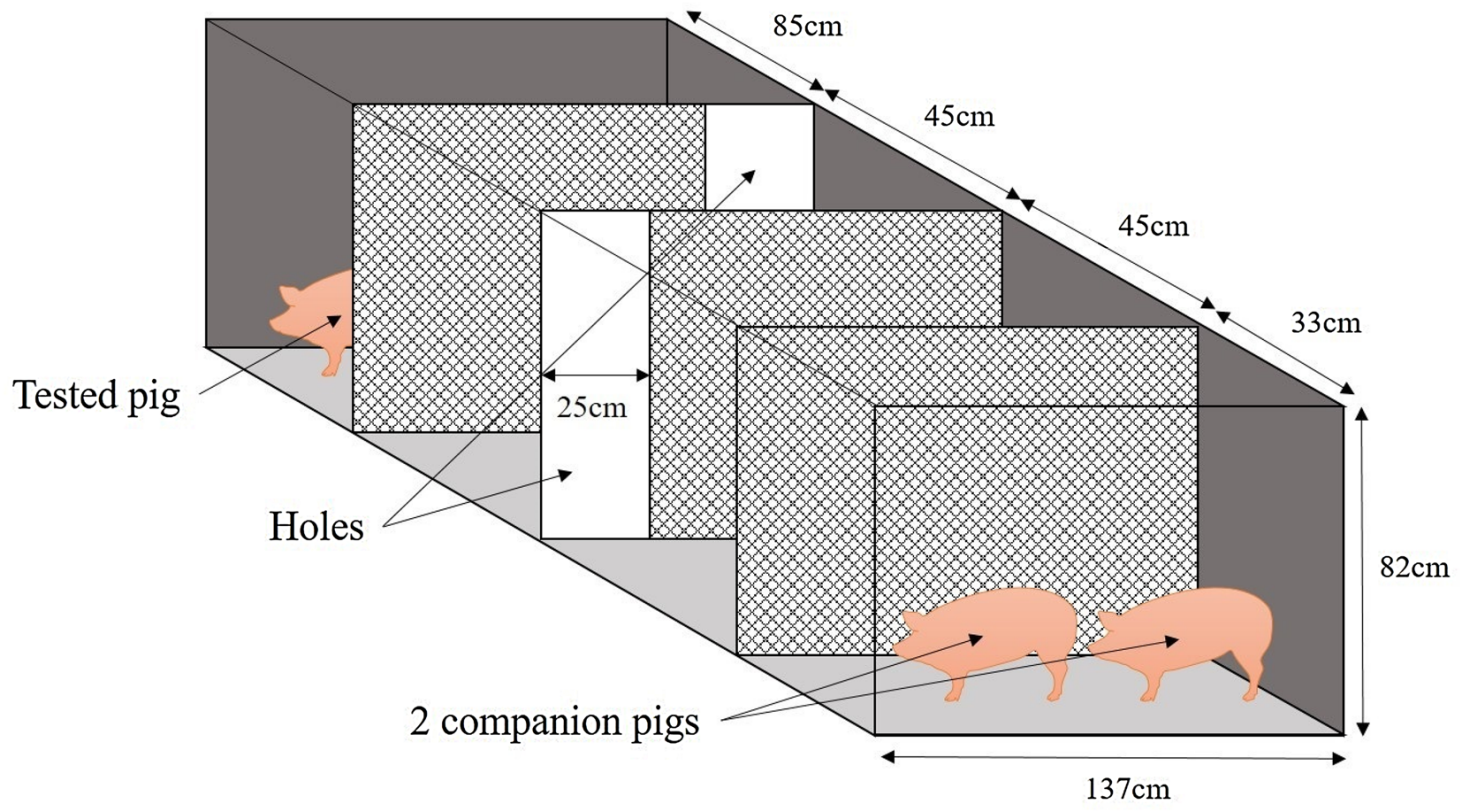
Schematic of the device used for the barrier test (BARR), with the tested pig starting on the opposite side of the two companion piglets.

This test was carried out only during afternoon between 15:00 h to 17:00 h in the habituation arena. The test consisted of a session of five successive trials. To succeed in reaching its siblings, the piglets had to negotiate a path through the two fences by going left and then right (Fig. 1). The piglet had a maximum of 6 min to accomplish the task. Each trial was stopped when the tested piglet touched one of the two companion piglets. At the end of each trial, the piglet was picked up and placed back at the same starting point on the opposite side of the arena from the companion piglets. All the handling of the tested piglet was carried out by the same familiar experimenter to avoid any supplemental stress. The test was videotaped and analyzed by a single trained observer blind to treatment using Ethovision XT 13^14^. The variables analyzed were as follows: total distance travelled, latency to cross the first hole (= front leg crossing) and the overall trial duration. One piglet was removed from the test because of lameness.

#### Spatial T-maze task (TMAZE)

A spatial T-maze task was used to evaluate spatial learning and memory from 33 to 41 days of age.

##### Familiarization with the reward

As this test is based on feed motivation, a 4-day training period was carried out to train the piglets to associate a bowl with a feed reward between 29 to 32 days of age, and also to maintain their comfort with being alone. The reward used was the same chocolate milk used to deliver the synbiotic. Piglets were trained in the habituation arena. The bowl (H9.5 × Ø15 cm) was embedded in a wood box for more stability, and attached to the wall.

The habituation to the bowl as container of a feed reward was carried out by a single experimenter with one or two sessions per day, separated at least by a 45-min break while piglets were brought back to their home pen, between 7:00 h to 19:00 h (random assignment to mornings or afternoons). Introduction of the piglets in the arena was always done at the same location of the arena, at the opposite side to the bowl, oriented towards the bowl. When the habituation sessions were carried out, the piglets were no longer being supplemented with the chocolate milk, and this helped maintain their motivation for the milk reward. During the whole process, the amount of milk was progressively reduced. Indeed, bigger volumes were easier to learn to drink in a bowl for the early habituation sessions but for the test it needed to be lowered to avoid satiety. The process followed a progressive pattern: day 1–15 min maximum for a single session, 250 ml of chocolate milk available; day 2–5 min maximum for two sessions, respectively 250 ml and 100 ml available; day 3–5 min then 3 min maximum, respectively 100 ml and 50 ml available; day 4–3 min maximum for two sessions, 30 ml available. All the piglets always managed to succeed before the maximum time allowed. The session stopped when they had drunk the total amount of milk or when they did not show any more interest in it. The variables recorded were the time needed to touch the bowl for the first time as well as the time to drink from the bowl for every trial.

##### Cognitive test

This test was adapted from Elmore et al.^11^. The T-maze arena was composed of 4 distinct arms (North, South, West and East) of similar dimensions L235 × W70 × H82 cm (see the Supplementary Figure in the Supplementary Information). Only 3 arms were used during each test (West, East, and either North or South). The unused arm was closed by a removable wall. The spatial visual patterns used for the test were made with tape and directly fixed on the walls of the arena (dots, waves, vertical and horizontal strips, respectively for North, South, West and East). The ends of the North and South arms were used as the starting boxes for the trials (two similar areas of L80 × W70 × H82 cm with a sliding fence at the blind ends); whereas the West and East arms, containing a bowl at their blind ends, were used as rewarded and unrewarded arms. The bowl of the rewarded arm was filled with 20 ml of chocolate milk, whereas the bowl of the unrewarded arm was empty and a cup filled of 20 ml of chocolate milk (unreachable by the piglets but providing the same odor cue) was positioned under it in order to prevent piglets from selecting an arm because of milk odor cues. The maze was cleaned between trials, and the experimenter went in both rewarded and unrewarded arms to ensure even spread of human odors and to prevent giving piglets any extra visual cues.

The T-maze test was composed of two distinct stages: a 6-day acquisition stage (A1–6) from 33 to 38 days of age during which the piglet learnt to associate one T-maze arm with the feed reward; followed immediately by a 3-day reversal stage (R1–3) from 39 to 41 days of age during which the rewarded arm was switched compared to the acquisition stage. For both acquisition and reversal stages, piglets did 10 successive trials per day, between 8:00 h to 18:30 h (random assignment to mornings or afternoons). The arm selected to start each trial (5 North and 5 South) was randomized between days, with no more than 2 successive times in the same starting box over the 10 trials pattern. The pattern drawn per day was similar for all the piglets. The objective of these changes was to make sure piglets are not conditioned to always turn left or right but to really use additional cues (visual, odorous) to determine the rewarded arm and to avoid any laterality bias. The rewarded arm (West or East) was also randomized between piglets. Once a piglet found the rewarded bowl, it was given 10 s to drink about 10 ml of chocolate milk. Before the next trial, any feces were cleaned with paper towels, 10 ml of milk was added in the rewarded bowl to refresh it and keep an approximate amount of 20 ml. The milk in the cup under the unrewarded bowl was refreshed between piglets. On A1 and A2 of the acquisition stage, piglets were allowed to go back and forth until they reached the rewarded bowl. The maximum time allowed per trial was 5 min. From A3 to A6 of the acquisition stage and from R1 to R3 of the reversal stage, piglets were no longer allowed to travel back and forth. Four options existed to stop a trial: (1) to select the right arm and drink milk for 10 s, (2) to select the right arm but turn back before reaching the rewarded bowl, (3) to select the wrong arm (piglet allowed to reach the end to check the bowl is empty), and (4) to do not choose any arm for the 60 s after the start of the trial. Video recordings were analyzed using the XP Observer 14 software (Noldus, The Netherlands) by a single trained observer blind to treatment. The variables analyzed were as follows: the proportion of correct choices per day, mean time to make a choice between the two arms (among the 10 trials per day), mean time to touch with its snout the rewarded bowl per day, minimum time to select the rewarded arm per day, number of trials needed to succeed for the first time in the reversal stage and number of trials needed to do two successful trials in the reversal stage. During the reversal stage, if a piglet never succeeded, the maximal 30 trial attempts was attributed.

Six piglets were excluded from this spatial test: 1 for lameness, 3 because of a lack of interest in the reward, 1 because of an error in the trials’ pattern on A2, and 1 that found handling to be highly aversive and was too stressed to perform the test. Of the remaining 26 piglets, 6 did not complete the reversal stage because of a too low success rate on A6 (less than 7/10 successful trials per day which can be due to a non-understanding of the task or a lack of motivation for the reward selected).

### Microbial analyses of feces

Samples for 16S rRNA gene composition analyses came from fresh feces collected at 16 days of age (during the SOR test) while still with the sow and at 33 and 41 days of age (during the first and last days of the T-maze test). Feces were put on ice and stored at − 80 °C until DNA extractions.

The DNA was extracted from 200 mg of frozen feces by bead beating using the Fast DNA SPIN kit for feces (MP Biomedicals Corporation, Irvine, CA, USA). Extracted DNA was then sent to the Argonne National Laboratory Environmental Sample Preparation and Sequencing Facility (Lemont, IL, USA) for PCR amplification of the V4 region of the 16S rRNA gene (515F-806R) (Forward: GTGYCAGCMGCCGCGGTAA; Reverse: GGACTACNVGGGTWTCTAAT) and sequenced using the MiSeq reagent kit V2 on an Illumina MiSeq (500 cycles) (Illumina Inc., San Diego, CA, USA). The sequencing library was generated using an integrated 12-base Golay barcode in the forward primer. Each 25 µL PCR reaction contains 9.5 µL of MO BIO PCR Water (Certified DNA-Free), 12.5 µL of QuantaBio’s AccuStart II PCR ToughMix (2 × concentration, 1 × final), 1 µL Golay barcode tagged Forward Primer (5 µM concentration, 200 pM final), 1 µL Reverse Primer (5 µM concentration, 200 pM final), and 1 µL of template DNA. The conditions for PCR were as follows: 94 °C for 3 min to denature the DNA, with 35 cycles at 94 °C for 45 s, 50 °C for 60 s, and 72 °C for 90 s; with a final extension of 10 min at 72 °C to ensure complete amplification.

### Sequence processing and microbial community analyses

Briefly, 16S rRNA gene sequences were processed and clustered using the mothur v.1.39.3 standard operating procedure (SOP) designed for MiSeq data^17^; the mothur MiSeq SOP (version 132) was accessed in August 2018. The SILVA-based bacterial reference alignment was used to identify the taxonomy of OTUs at a cluster cutoff of 97% sequence identity. Measures of richness, evenness and diversity were determined in mothur. Richness can be defined as the number of OTUs observed per sample, evenness represents the uniformity of the distribution of OTUs amongst a community across the multiple observed OTUs, while α-diversity is a concept combining both richness and evenness^18^. Richness and α-diversity were determined through the coverage, the number of OTUs observed, the Chao1, the ACE, the Shannon, the Simpson, the inverse Simpson estimators and β-diversity metrics were calculated using the Yue and Clayton’s theta metric (thetaYC, as implemented in mothur) distances^19^. The OTU table was rarefied to a minimum of 1000 sequences per sample to account for differences in sampling effort. A linear model with the treatment (CTL, SYN) and the day of sampling (D16, 33 and 41) was used for the richness, evenness and diversity analyses. The effects of dietary treatments and time periods on the microbial community structure were tested using an analysis of molecular variance (AMOVA) of the thetaYC distance matrix in mothur. Results were adjusted by using the Bonferroni correction. The “metastats" command in mothur was then used to determine the OTUs responsible for the significant differences observed using AMOVA. The “corr.axes” and “otu.association” commands in mothur, specifying the default Pearson method, were then used in combination to estimate the significant Pearson correlations between behavioral indicators and bacterial populations.

### Statistical analysis

Statistical analyses were performed with the software R 3.4.3^20^. The variables of time to drink during the habituation to the bowl of TMAZE, the BARR test variables, as well as the mean times to make a choice and to touch the rewarded bowl were normalized by logarithmic transformation before statistical analysis. Other variables were normal without transformation. In all the statistical analysis, p < 0.05 was considered statistically significant and 0.05 ≤ p < 0.1 as a trend.

Linear model with the treatment as fixed effect was used to test the time for the piglets to touch the bowl the first time during habituation to the TMAZE test, as well as for the variables of the SOR test. For the familiarization phase of the SOR test, the fixed effect was also replaced by the object type and the arena of the test. A mixed effects model for repeated measures with the treatment, the trial or the day and the interaction between the two factors as fixed effects and the animal being included as random effects were used to test the effect of habituation variables to isolation and the TMAZE bowl, as well as the barrier test and the TMAZE test variables. The statistical unit for the previous traits was the animal. These analyses were done with the function lmer from the R package “lme4” 1.1-7. The emmeans function from the R package “emmeans” 1.2-2 was used to perform pairwise comparisons with the FDR correction when interactions were significant (p < 0.05). A generalized linear model (family: Poisson, link: log) was used to analyze the number of trials needed to succeed in the reversal stage of the T-maze, with the treatment as fixed effect and the side of the rewarded arm as a random effect. This analysis was done with the function glmer from the R package “lme4”.

## Results

Means, SEM of untransformed traits from the SOR, BARR and TMAZE tests are reported in Table 1. Piglets had similar weight at weaning (CTL: 14.5 ± 2.0 kg; SYN: 13.1 ± 2.8 kg; p > 0.1).

**Table 1:** Numbers, means, SE of untransformed traits in the SOR (Spontaneous Object Recognition), the BARR (Fence barrier task) and the TMAZE (Spatial T-maze task) for Controls (5 ml TruMoo^®^ Chocolate—Whole milk) and Synbiotic (3 strains of *Lactobacillus* at 10^9 CFU^/piglet, fructo-oligosaccharide at 10 mg/day/piglet, beta-glucan at 11 mg/day/piglet, vitamin C at 10 mg/day/piglet diluted in 5 ml of chocolate milk) treated piglets.

Test	Traits^A^	Trial or day	Controls		Synbiotics		Treatment	Trial/day	Treatment × trial/day
			N	Mean ± SE	N	Mean ± SE	p value	p value	p value
SOR	Lat. to interact with the old object (s)		14	283 ± 38.8	13	201 ± 34.9	0.13		
	Total Dur. of interaction with the old object (s)		18	14.6 ± 3.7	17	20.7 ± 5.2	0.34		
	Lat. to interact with the new object (s)		16	255 ± 30^b^	14	165 ± 31^a^	0.046		
	Total Dur. of interaction with the new object (s)		18	27.3 ± 7.0	17	27.4 ± 6.1	0.99		
	Freq. in the zone of the old object (n)		18	4.0 ± 1.2^a^	17	7.8 ± 1.2^b^	0.030		
	Freq. in the zone of the new object (n)		18	5.2 ± 1.2	17	7.6 ± 1.2	0.16		
	Discriminant Index		17	0.30 ± 0.13	14	0.22 ± 0.14	0.69		
BARR	Total distance travelled (m)	1	16	11.9 ± 1.6	15	9.7 ± 1.4	0.16	< 0.0001	0.027
		2	16	6.4 ± 1.3	16	6.3 ± 0.6			
		3	16	7.4 ± 1.2^b^	15	4.2 ± 0.3^a^			
		4	17	5.3 ± 0.7	15	3.8 ± 0.2			
		5	17	4.7 ± 0.5	16	4.9 ± 0.8			
	Lat. to cross the 1st hole (s)	1^a^	16	61.9 ± 8.9	16	67.3 ± 10.4	0.82	< 0.0001	0.43
		2^b^	15	15.0 ± 1.9	16	24.9 ± 4.3			
		3^b^	16	27.9 ± 8.5	16	18.0 ± 3.3			
		4^b^	17	25.8 ± 7.4	16	29.4 ± 15.1			
		5^b^	17	22.3 ± 5.1	16	25.3 ± 6.5			
	Trial duration (s)	1^a^	15	96.3 ± 17.6	14	93.3 ± 12.2	0.78	< 0.0001	0.23
		2^b^	15	25.1 ± 2.8	16	37.4 ± 4.5			
		3^b^	15	40.2 ± 9.4	15	25.1 ± 2.5			
		4^b^	16	38.1 ± 10.3	15	23.3 ± 3.7			
		5^b^	17	30.8 ± 5.3	16	36.7 ± 11.4			
TMAZE	Mean duration to touch the rewarded bowl (s)	A1^v^	13	52.3 ± 9.6	11	48.3 ± 7.9	0.12	< 0.0001	0.33
		A2^v^	13	40.1 ± 7.5	11	47.6 ± 6.1			
		A3^u^	12	24.3 ± 5.6^b^	11	13.0 ± 1.9^a^			
		A4^u^	12	13.2 ± 2.0	11	17.4 ± 2.3			
		A5^u^	13	11.9 ± 1.4	11	14.1 ± 2.5			
		A6^u^	13	15.9 ± 3.3	11	13.9 ± 2.3			
		R1^z^	7	30.1 ± 7.4	7	22.9 ± 3.7			
		R2^y^	8	12.2 ± 2.5	9	18.5 ± 3.1			
		R3^y^	10	12.9 ± 3.0	9	19.8 ± 4.4			
	First trial to try the new rewarded arm (n)	R1–3	13	19.8 ± 4.3^t^	11	10.3 ± 3.3^s^	0.048		
	First two successive trials during the reversal stage (n)	R1–3	13	23.7 ± 3.9	11	17.7 ± 3.9	0.19		

Statistical models used: (1) traits from the SOR test: a linear model Y ~ Treatment; (2) traits from the BARR test: a linear mixed model for repeated variables Y ~ Treament + Trial + Treatment × Trial + (1|pig); (3) traits from the TMAZE test: for durations, a linear mixed models for repeated variables Y ~ Treament + Day + Treatment × Day + (1|pig) and, for number of trials generalized linear mixed model following a Poisson law Y ~ Treatment + (1|side of the reward).

Letters were attributed per test in alphabetical order for significantly different values (p < 0.05), regarding both the trial/day and the treatment effect. Discriminant Index: (DN1: Duration interacting with the novel object − DO3: Duration interaction with the old object)/(DN1 + DO3). Lat: Latency; Freq: Frequency; Dur: Duration.

^A^A3 to A6: days 3 to 6 during the acquisition stage of the test; R1 to R3: days 1 to 3 during the reversal stage of the test.

### Habituation to the arena

There were no interactions between treatment and trial or treatment effects on all habituation variables (p > 0.1). However, there was a trial effect on the distance travelled (p < 0.001) with a significant decrease from the first two trials alone in comparison to the three last ones, on the latency to jump for the first time (p < 0.001) and on the number of jumps (p = 0.039) with a decrease from the first trial alone compared with the other four trials. There was also a tendency toward a decrease in the number of times entering the peripheral area (p = 0.060) from the first trial alone compared with the other four trials, and no effect on the percentage of time spent near the periphery (p > 0.1).

### Spontaneous object recognition test

During familiarization, there was no effect of the object used, the test arena or the treatment on the number of interactions (6.5 ± 4.7 times) or the total duration of interactions (25.9 ± 23.8 s) with both objects. There were also no effects on number of times entering the designated area near the objects (7.0 ± 5.6 times) or the total duration of time spent in the designated area (60.3 ± 44.2 s, both p > 0.1).

**Figure 2:**
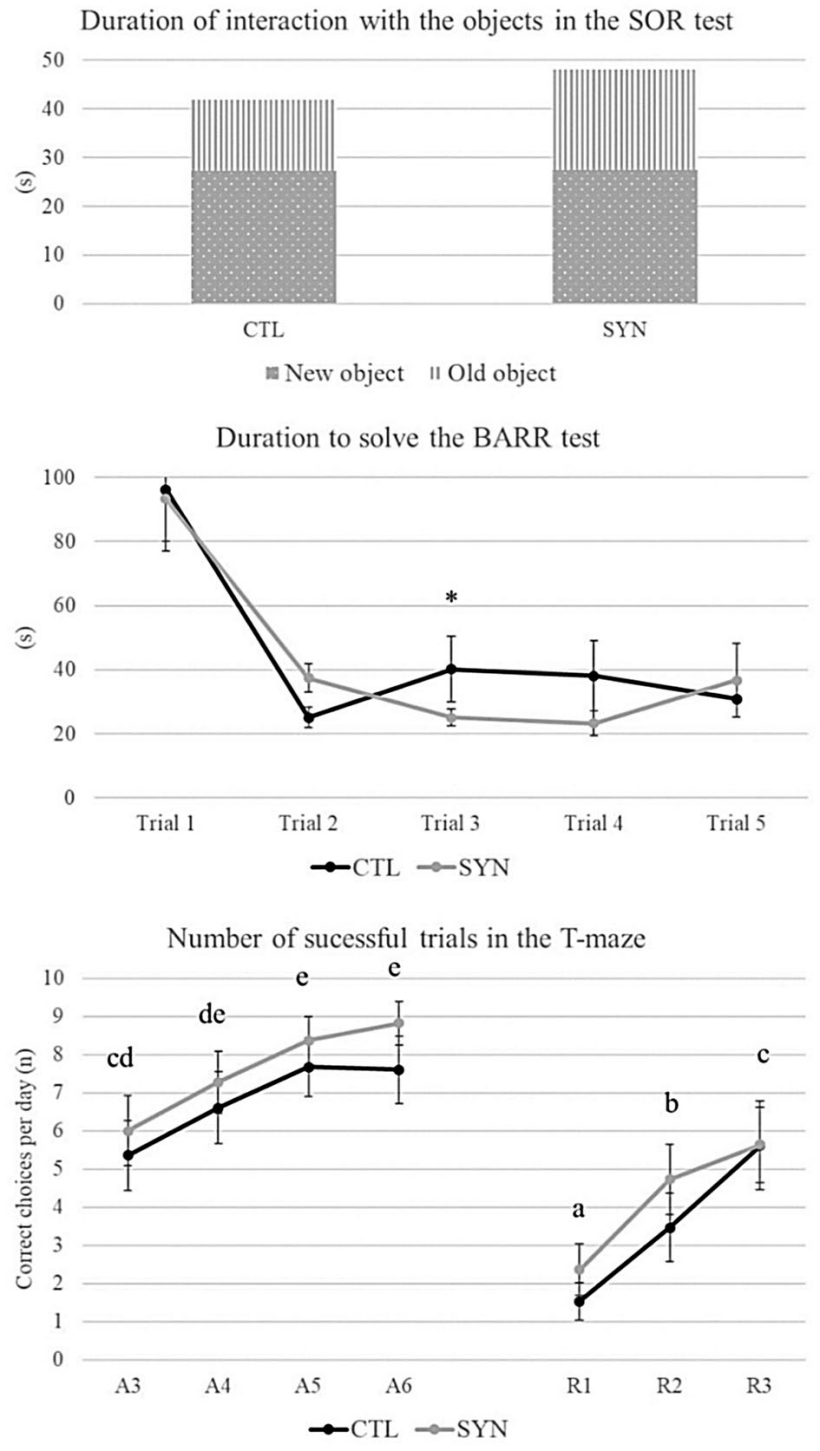
Cognitive performances of the pigs of the two treatment diets (CTL: Controls, 5 ml TruMoo^®^ Chocolate—Whole milk versus SYN: Synbiotics, 3 strains of *Lactobacillus* at 10^9 CFU^/piglet, fructo-oligosaccharide at 10 mg/day/piglet, beta-glucan at 11 mg/day/piglet, vitamin C at 10 mg/day/piglet diluted in 5 ml of chocolate milk). ^1^Letters differ at p < 0.05 between days; *p < 0.05. ^2^A: trials during the acquisition phase; R: trials during the reversal phase.

During the novelty stage, the side of introduction of the new object, as well as the type of object had no effect on all variables recorded (p > 0.1). SYN piglets visited the area with the old object more than CTL piglets (7.8 ± 1.6 times vs. 4.0 ± 0.7 times, p = 0.030). However, their latency to touch the new object was shorter than CTL piglets (see Table 1, p < 0.05). There was no effect of the treatment group on the DI (p > 0.1; Fig. 2).

### Barrier test

The analysis of the variables showed an interaction between treatment and trial for the total distance (p = 0.027) with CTL piglets travelling further than SYN piglets on trial 3 (see Table 1). There was an effect of the trial on all other variables (p < 0.001) with a decrease from trial 1 over the 4 other trials, but no other effects of treatment supplementation on test variables (see Table 1 and Fig. 2, p > 0.1).

### T-maze test

During the habituation to the bowl as a reward for the T-maze, there was no interaction effect between the treatment and trial. Piglets needed less time to drink in the bowl over trials (p < 0.001). They needed 153.4 ± 149.5 s on the trial 1 and only 7.0 ± 7.6 s on trial 4. Overall, SYN piglets took more time 38.6 ± 88.4 s to drink than CTL piglets 25.4 ± 63.5 s (p = 0.024). They took the same time to touch the bowl for the very first time 59.1 ± 70.5 s (p > 0.1).

During the test, there were no interactions between treatment and day regarding the number of correct choices per day (p > 0.1). Treatment effect was not significant (p > 0.1) but the day of test was (p < 0.001) with a clear progression within both phases (Fig. 2). On A3, SYN piglets needed less time to touch the rewarded bowl than CTL piglets (13.0 ± 1.9 versus 24.3 ± 5.6 s, p = 0.03).

During the reversal stage, the SYN piglets needed fewer trials to find the reward in the new arm than CTL piglets (10.3 ± 3.3 trials versus 19.8 ± 4.3, p = 0.048).

### Correlations between cognitive traits

Pearson correlations between the durations from the different cognitive task were summarized in a Supplementary Table 1. The three different tests were correlated together and showed medium to high correlations from 0.32 to 0.76 (p < 0.1).

### Microbiota analyses

#### Richness, diversity and composition of microbiota

The richness and diversity index of the bacterial community varied with Time only (16, 33 or 41 days of age) and did not vary with treatment (Table 2). The richness, evenness and diversity of gut communities were highest on day 33. The average number of OTUs observed per sample was 150 ± 4, resulting in coverages of 99.0 ± 0.1%.

**Table 2:** α-diversity indicators of the microbiota in feces samples, at three different days (D16, D33 and D41) after the beginning of two different treatment diets: Controls (5 ml TruMoo^®^ Chocolate—Whole milk) versus Synbiotic (3 strains of *Lactobacillus* at 10^9^ CFU/piglet, fructo-oligosaccharide at 10 mg/day/piglet, beta-glucan at 11 mg/day/piglet, vitamin C at 10 mg/day/piglet diluted in 5 ml of chocolate milk).

Traits	Treatment			Day			
	Controls	Synbiotics	p value	16	33	41	p value
Chao1 estimator	246.2 ± 7.6	248.1 ± 7.8	> 0.1	184.7 ± 8.7a	295.2 ± 9.6b	261.5 ± 9.8b	1.4e−12
ACE estimator	292.6 ± 9.4	299.9 ± 9.6	> 0.1	211.9 ± 10.8a	359.9 ± 11.9c	316.9 ± 12.2b	4.4e−14
Shannon index	3.91 ± 0.06	3.90 ± 0.06	> 0.1	3.70 ± 0.07a	4.16 ± 0.08b	3.86 ± 0.08a	1.0e−04
Simpson index	0.050 ± 0.004	0.050 ± 0.004	> 0.1	0.053 ± 0.005a	0.037 ± 0.005a	0.058 ± 0.005b	0.013
Inverse Simpson index	27.3 ± 2.0	26.1 ± 2.1	> 0.1	23.4 ± 2.3a	32.8 ± 2.6b	23.7 ± 2.6a	0.015
Species observed	151.9 ± 4.8	151.9 ± 5.0	> 0.1	118.7 ± 5.6a	176.7 ± 6.2b	160.4 ± 6.3b	1.4e−9
Coverage (%)	99.1 ± 0.2	98.9 ± 0.2	> 0.1	99.4 ± 0.2	99.0 ± 0.2	98.7 ± 0.2	0.072

Statistical linear model formula: Trait ~ Treatment + Time. Letters were attributed for significantly different values a < b < c.

Adjusted means ± SEM.

Three different phyla were found across all samples: *Firmicutes* represented 70.9% of the total sequenced DNA, *Bacteroidetes* 19.2% and *Proteobacteria* 3.2%. All the remaining phyla represented less than 3%. The major classes of the *Firmicutes* phylum were *Clostridia* (60.7% of the sequences), *Bacilli* (20.6%) and *Negativicutes* (12.0%); of the *Bacteroidetes* phylum were *Bacteroidia* (88.2%) and *Bacteroidetes unclassified* (11.8%); of the *Proteobacteria* phylum were *Gammaproteobacteria* (44.0%), *Deltaproteobacteria* (30.4%), *Epsilonproteobacteria* (17.0%) and *Betaproteobacteria* (5.5%).

#### Effects of treatments and day of sampling on microbiota composition

**Figure 3:**
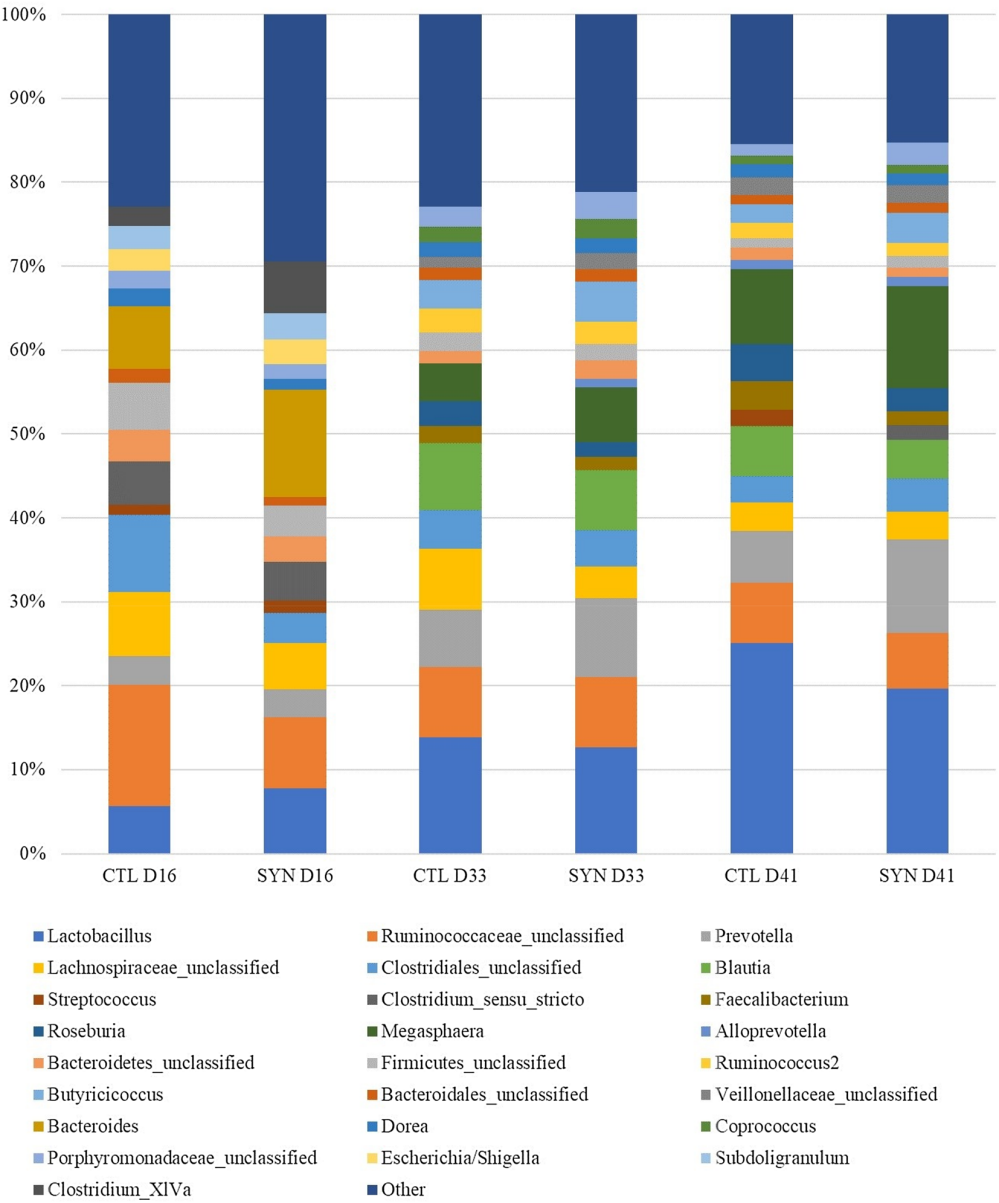
Relative abundances of genera for the two supplementation treatments (CTL: Controls, versus SYN: Synbiotics) on three days of sampling (D16, D33 and D41). Genera outside the 25 most abundant are combined as “Other.”

**Figure 4:**
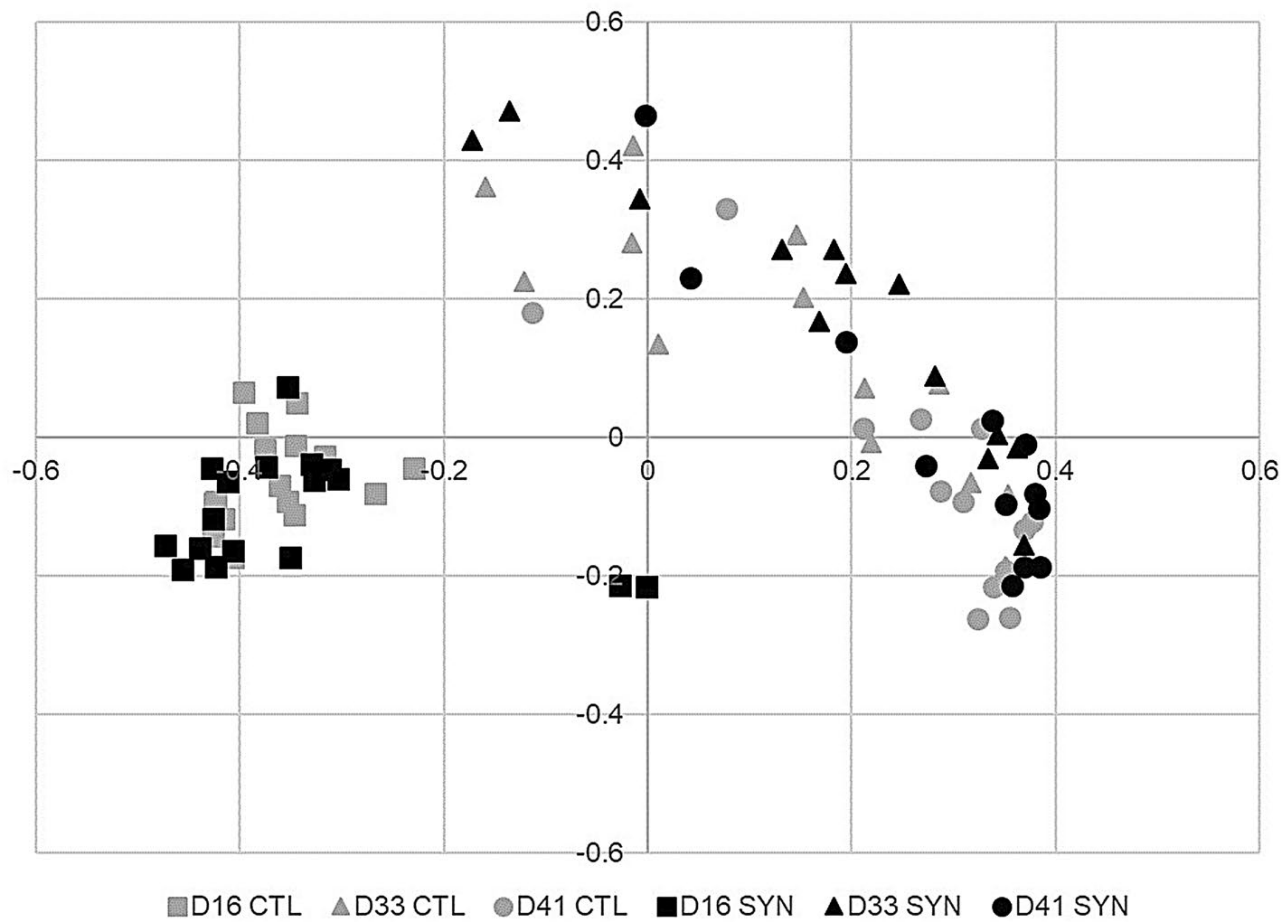
β-diversity of gut communities with respect to supplementation treatments and day of sampling. Distances were calculated using the Yue and Clayton theta metric and plotted using PCoA for the two supplementation treatments (CTL: Controls versus SYN: Synbiotics) on three day of sampling (D16, D33 and D41).

**Figure 5:**
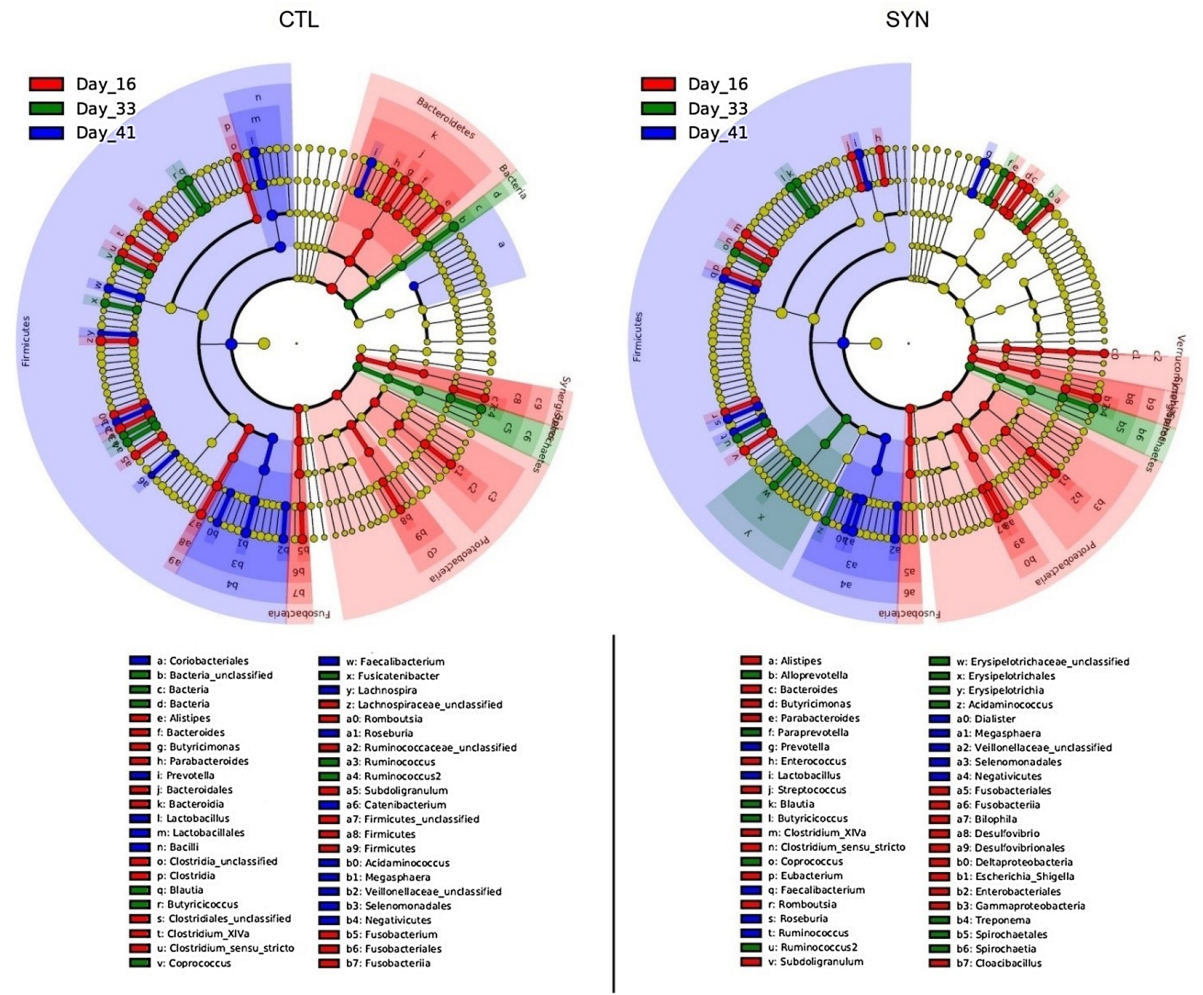
Linear discriminant analysis of taxa differentiating the pigs of the two supplementation treatments (CTL: Controls versus SYN: Synbiotics) on three day of sampling (D16, D33 and D41). Taxa with LDA scores > 3.5 as computed via LEfSe are plotted on the cladograms. Unclassified taxa are referenced as “uncl.”

The microbial community structure and its change over time is graphically presented with respect to relative abundances of genera (Fig. 3), β-diversity (Fig. 4) and taxa found to be linear discriminants of CTL and SYN treatments (Fig. 5). The effects of supplementation treatments and time of sampling are presented in Table 3. Across treatments, β-diversity did not reveal significant clustering of overall community structure by treatment, but within each treatment group, the microbiota composition was different over time (p < 0.05). Interestingly, linear discriminant analysis revealed that the succession of gut microbiota over time varied across CTL and SYN treatment. At day 16, CTL and SYN piglets had similar microbiota composition (p > 0.1). However, some taxa were specific to a treatment diet. SYN piglets had *Campylobacter*, *Fusobacteirum*, *Cloacibacillus*, *Eubacterium*, *Fusobacteriaceae* and *Akkermansia* while CTL had not; while CTL had *Romboutsia* and *Alistipes*. At day 33, there was a tendency for CTL microbiota composition to be different from SYN composition (p = 0.066). In contrast, SYN piglets had *Alloprevotella*, *Erysipelotrichaceae*; while CTL piglets had *Holdemanella*, *Phascolarctobacterium* and *Fusicatenibacter*. At day 41, CTL and SYN piglets had significantly different microbiota composition (p = 0.047) with more *Prevotella*, *Lachnospiraceae* and *Ruminococcaceae* for CTL piglets. There was a tendency for *Firmicutes/Bacteroidetes* ratio to be higher in CTL group than in SYN group (p = 0.079) and also higher at day 41 in comparison to day 16 (p = 0.062). The treatment had no effect on the percentage of *Actinobacteria* or *Proteobacteria* (p > 0.1). However, SYN piglets had a higher percentage of *Bacteroidetes* (p = 0.017) and a lower percentage of *Firmicutes* (p = 0.012) than CTL piglets. The presence of *Clostridium *sensu stricto and *Treponema* was specified to SYN piglets; while CTL piglets had *Streptococcus* and SYN piglets had not. The percentage of *Actinobacteria* did not change over time (p > 0.1), whereas percentage of *Bacteroidetes* was lower at days 33 and 41 in comparison to day 16 (p = 0.014 and p = 0.011, respectively), the percentage of *Firmicutes* was also lower at days 33 and 41 in comparison to day 16 (p = 0.0070 and p = 0.0028, respectively) and day 33 was lower than day 41 (p = 0.033). The percentage of *Proteobacteria* decreased between day 15 to days 33 and 41 (p = 0.020 and p = 0.015, respectively).

**Table 3:** Effects of two different treatment diets (CTL: Controls, 5 ml TruMoo^®^ Chocolate—Whole milk versus SYN: Synbiotics, 3 strains of *Lactobacillus* at 10^9 CFU^/piglet, fructo-oligosaccharide at 10 mg/day/piglet, beta-glucan at 11 mg/day/piglet, vitamin C at 10 mg/day/piglet diluted in 5 ml of chocolate milk) and day of sampling (D16, D33 and D41) on 16S rRNA gene microbiota composition.

Treatment × day				p value	Bacteria taxa for significant treatment × day interactions (*Genus level*)^a^	
CTL	16	SYN	16	NS		
CTL	33	SYN	33	0.066		
CTL	41	SYN	41	0.047	*Prevotella* *Lachnospiraceae unclassified* *Ruminococcaceae unclassified*	CTL_D41 > SYN_D41 CTL_D41 > SYN_D41 CTL_D41 > SYN_D41
CTL	16	CTL	33	< 0.001	*Actinobacteria* *Bacteroidetes* *Elusimicrobia* *Firmicutes* *Lentisphaerae* *Proteobacteria* *Spirochaetes*	CTL_D16 > CTL_D33 CTLD16 < CTL_D33 CTL_D16 > CTL_D33 CTL_D16 > CTL_D33 CTLD16 < CTL_D33 CTL_D16 > CTL_D33 CTL_D16 > CTL_D33
CTL	16	CTL	41	< 0.001	*Actinobacteria* *Bacteroidetes* *Chlamydiae* *Fibrobacteres* *Firmicutes* *Proteobacteria* *Spirochaetes*	CTL_D16 > CTL_D41 CTL_D16 < CTL_D41 CTL_D16 > CTL_D41 CTL_D16 > CTL_D41 CTL_D16 > CTL_D41 CTL_D16 < CTL_D41 CTL_D16 > CTL_D41
CTL	33	CTL	41	0.012	*Porphyromonadaceae unclassified* *Prevotella* *Fibrobacter* *Streptococcus* *Blautia* *Ruminococcaceae unclassified* *Treponema*	CTL_D33 < CTL_D41 CTL_D33 < CTL_D41 CTL_D33 > CTL_D41 CTL_D33 > CTL_D41 CTL_D33 < CTL_D41 CTL_D33 < CTL_D41 CTL_D33 < CTL_D41
SYN	16	SYN	33	< 0.001	*Actinobacteria* *Bacteroidetes* *Firmicutes* *Fusobacteria* *Proteobacteria* *Spirochaetes* *Synergistetes*	SYN_D16 > SYN_D33 SYN_D16 < SYN_D33 SYN_D16 > SYN_D33 SYN_D16 < SYN_D33 SYN_D16 < SYN_D33 SYN_D16 > SYN_D33 SYN_D16 < SYN_D33
SYN	16	SYN	41	< 0.001	*Actinobacteria* *Bacteroidetes* *Firmicutes* *Proteobacteria* *Spirochaetes* *Synergistetes*	SYN_D16 > SYN-D41 SYN_D16 < SYN_D41 SYN_D16 > SYN-D41 SYN_D16 < SYN_D41 SYN_D16 > SYN-D41 SYN_D16 < SYN_D41
SYN	33	SYN	41	0.025	*Porphyromonadaceae unclassified* *Lachnospiraceae unclassified*	SYN_D33 < SYN_D41 SYN_D33 > SYN_D41

Analysis of molecular variance (AMOVA) using the standardized distance matrix method in mothur adjusted by using Bonferroni correction. The determination of the bacteria responsible for the AMOVA significant differences were analyzed with the command “metastats" in mothur from the mothur standard operating procedure (SOP) designed for MiSeq data^17^. The mothur MiSeq SOP was accessed in August 2018.

^a^Bacterial taxa mentioned had a p-value below 0.001.

#### Relation between bacteria taxa and behavioral traits

The duration to succeed in trial 2 of the BARR test was the only trait significantly associated with bacterial populations (p = 0.05; Supplementary Table 2). The trait had a negative medium Pearson correlation (r = − 0.32) with a *Bacteroidetes*, *Prevotella*, and medium positive correlations with different *Firmicutes*: *Clostridium XIVa* and *XVIII, Faecalicoccus* and *Ruminococcaceae* (respectively r = 0.31, 0.35, 0.31 and 0.36).

## Discussion

In livestock, there is a gap of knowledge about the potential effects of feed additives on general behaviors and even more when looking at effects on learning and memory processes. When using a specific feeding strategy, probiotics, prebiotics or synbiotics, the postulated hypothesis is that the supplementation will affect the gut microbiota composition. Indeed, diet and feed additives are powerful tools to modulate the gut microbiota composition^2,21^. Those alterations of the gut microbiota composition are increasingly understood to affect brain and cognitive functions through the gut-brain axis^2^. Many recent reviews about changes in gut microbiota composition, especially with probiotics and prebiotics, realized in rodents and humans have raised awareness about the impact of feeding on stress^22,23^, anxiety^24^, mood^25^, social behavior^26,27^ and cognition^28,29^. Looking especially at cognition, studies in rodents have demonstrated effects on working, spatial and non-spatial memories. In pigs, few studies have confirmed that feeding can impact learning and memory^30–33^. However, none of the studies in livestock looked at the repercussions of the feed on the gut microbiota composition, as a potential explanation for changes in behaviors and cognitive functions.

Memory is also sensitive to stress, especially chronic stress^34^. Stress can also change microbiota composition^35^ through, for example, modification of gut permeability^36–38^. Specific feeding strategies, in pigs, also demonstrated beneficial effect in terms of stress and fearful emotions using a large range of feed supplementation: vitamin E^39,40^, magnesium^41^, tryptophan^42–45^, aromatic plant extracts^40,46^, chitosan^47^, and the ratios of fat, cholesterol, carbohydrate^48^ and linoleic acid in the diet^49^. The use of feed additives can then be beneficial for both stress and memory abilities in pigs, but still remains an untapped solution to ameliorate problem of welfare in swine production.

In the current study, female piglets supplemented with the synbiotic demonstrated some improved aspects of memory in certain tests. They showed some improved working memory performance in a fence barrier task test, were quicker to learn during the acquisition stage of a T-maze solving task and showed higher learning flexibility during the reversal stage of the T-maze. It confirmed previous findings about effects on working and reference memories in pigs with other feeding supplements: high fat or sugar diets^31,33^ and dietary sialic acid^30,32^. Both high fat or sugar diets impaired working and reference memories of male Gottingen minipigs^31^. However, exposition of piglets for 8 weeks prior to birth to a high fat and high sugar diet showed improved working and reference memories^33^. In the two studies mentioned, a potential mechanism, proposed by the authors, to explain the diets’ effects on memory can be through the cholesterol level. Indeed, cholesterol is involved in synapse formation and synaptic structural plasticity of brain cell membranes, important for the development of memory abilities^50,51^. The brain may require a supply in triglycerides within a range to allow optimal development. Dietary sialic acid supplementation improved the performance of piglets in an 8-arm radial maze^30^ and in a T-maze^32^. Sialic acid is involved in neurogenesis^52^, neural repair and learning and memory^53^. The only study about supplementation with a probiotic for livestock was found in poultry. Supplementation of 1-day old Japanese quail for 36 days with the probiotic *Pediococcus acidilactici* improved their spatial memory in a holeboard-like test^54^.

Pigs in the current study were supplemented from 24-h after birth until 28 days. The early-life period is of high sensitivity for the organism, changes that occurs during that period can change the structure and the development of organs, as the brain^55,56^. Therefore, alterations of gut microbiota in early-life, with a severe stress or supplementation can produce long-term repercussions on the animal development^57^. Piglets from the SYN and CTL tended to differ in gut microbiota composition on day 33 and differed on day 41. Supplementation stopped on day 28, yet gut microbiota remained divergent 5 to 13 days later, suggesting that the synbiotic orally administrated daily may exert influence even after feeding has stopped^57^. The differences of gut microbiota composition between SYN and CTL piglets is a potential explanation to justify the difference of memory abilities between the two treatment groups. Indeed, several pathways of communication between the gut and the brain exist: through the neuronal, endocrine or immune systems^2,58–60^. In rodents, it has been demonstrated that alterations of gut microbiota were correlated with changes in brain-derived neurotropic factor and c-fos proteins concentrations, both known for their implications in memory functions^61–63^. In pigs as well, supplementation for a 28-day period with a higher crude protein diet showed an increase in dopamine concentration in the brain stem^64^. Probiotics can modulate the two main actors of gut-brain communication: cytokines^65^ and tryptophan, a precursor of serotonin, a brain neurotransmitter^66^. It was also showed that microbiota composition changes can modulate levels of brain neurotransmitters, as dopamine and serotonin^67^ involved in cognitive functions^68^. SYN treatment did not influence overall diversity of the gut microbiota, suggesting that community richness and evenness were not likely related to SYN mechanisms of influence on behaviour. Interestingly, although differences among CTL and SYN animals were modest when compared between groups at each time point, CTL and SYN microbiomes displayed distinct successional trajectories. Our data suggest that microbiota composition on day 33 tended to be different between the two treatment groups, and these developed to significantly different communities by day 41; however, the paths by which the CTL and SYN microbiomes reach these differences are also distinct. For example, *Paraprevotella* and *Alloprevotella* are linear discriminants of SYN but not CTL microbiomes on Day 33, though the mechanistic drivers and functional outcomes of this are unknown. Improvements in terms of memory in the present study were already visible on day 15 while the microbiota composition analysis between the two experimental groups was not significantly different. The most likely explanation for the absence of microbiota distinction on day 15 while cognitive effects were already observed may be due to observation of microbiome structures through 16S DNA analyses of fecal samples. Feces are a mirror of the real gut microbiota composition but still differ from it^69^. The difference induced by the supplementation may have been too subtle to be detectable in the fecal samples but already present in the gut. Moreover, alterations in microbial function in the gut may, through the production or consumption of different metabolites, influence host physiology before the differences in abundances (which require significant growth to become apparent) manifest.

Specific bacteria have already been identified with a particular interest for their effects on cognitive functions. In rodents, *Mycobacterium vaccae*, a commensal bacterium, is as a modulator of cognition, acting both through the immune and serotonergic systems^70–72^. In the present study, this specific bacterium did not differ between the two experimental groups. In humans, high levels of *Bifidobacteria* and *Bacteroidetes* species were correlated with improvements in working memory^73^. SYN piglets had higher cognitive abilities and a higher percentage of *Bacteroidetes* in comparison to the CTL piglets. We also demonstrated that the total duration on the second trial of the BARR test was the only cognitive trait significantly correlated with specific bacterial populations, in that case phylum-level categorizations of *Bacteroidetes* and *Firmicutes* spp. When looking at general behavioral traits instead of memory traits, the number of significant correlations between traits and bacterial populations is rather high^27^. The lack of correlations between cognitive traits and bacteria may suggest a higher complexity and number of intermediates in the communication between the gut and learning/memory functions.

The current study also evaluated different types of memories (episodic, working and spatial) on the same individuals at different time points, what makes possible comparisons between different types of memories and within a memory overtime. Pigs spending more time interacting with the new object in the SOR had the worse working memory both in BARR and TMAZE tests. The correlations estimated in the present pigs supported studies done in humans, where it has been demonstrated that similar frontal regions are involved in both working memory and episodic memory processes^74^. Pigs paying more attention to the familiar object in the SOR test also needed more trials to succeed two successive times during the reversal stage of the TMAZE test. The number of trials needed to succeed two successive times during the reversal stage might be associated with a more flexibility in learning abilities. Working memory performances at days 15 during the SOR test and 33 during the TMAZE test were correlated, showing a stability of working memory performance over time.

## Conclusion

The supplementation of female piglets from 24-h after birth to 28 days of age with a synbiotic appeared to confer advantages in two of the three distinct cognitive tasks, regardless of the nature of the reward (social or food) and the type of memory requested (working and/or spatial). Oral supplementation in early life for 28 days may result in sustained change in gut microbiota composition. Performance in a specific memory task seemed to be predictive of performance of the individual over time with the same memory or in other tasks requested different type of memory.

## Supplementary Information

Supplementary Figures.

Supplementary Table 1.

Supplementary Table 2.

## Data Availability

The microbiota data analyzed in this study can be accessed at https://www.ncbi.nlm.nih.gov/bioproject/PRJNA607434.

## References

[1] Nawroth C (2019). Farm animal cognition: Linking behaviour, welfare and ethics. Front. Vet. Sci..

[2] Cryan JF, Dinan TG (2012). Mind-altering microorganisms: The impact of the gut microbiota on brain and behaviour. Nat. Rev. Neurosci..

[3] O'Mahony SM, Clarke G, Borre YE, Dinan TG, Cryan JF (2015). Serotonin, tryptophan metabolism and the brain–gut–microbiome axis. Behav. Brain Res..

[4] Isaacson R, Kim HB (2012). The intestinal microbiome of the pig. Anim. Health Res. Rev..

[5] Frese SA, Parker K, Calvert CC, Mills DA (2015). Diet shapes the gut microbiome of pigs during nursing and weaning. Microbiome.

[6] Campbell JM, Crenshaw JD, Polo J (2013). The biological stress of early weaned piglets. J. Anim. Sci. Biotechnol..

[7] Nabuurs MJ, van Zijderveld FG, de Leeuw PW (1993). Clinical and microbiological field studies in The Netherlands of diarrhoea in pigs at weaning. Res. Vet. Sci..

[8] Fairbrother JM, Nadeau E, Gyles CL (2005). *Escherichia coli* in postweaning diarrhea in pigs: An update on bacterial types, pathogenesis, and prevention strategies. Anim. Health Res. Rev..

[9] Dowarah R, Verma AK, Agarwal N (2017). The use of *Lactobacillus* as an alternative of antibiotic growth promoters in pigs: A review. Anim. Nutr..

[10] Gifford AK, Cloutier S, Newberry RC (2007). Objects as enrichment: Effects of object exposure time and delay interval on object recognition memory of the domestic pig. Appl. Anim. Behav. Sci..

[11] Elmore M, Dilger R, Johnson R (2012). Place and direction learning in a spatial T-maze task by neonatal piglets. Anim. Cogn..

[12] Moustgaard A, Lind NM, Hemmingsen R, Hansen AK (2002). Spontaneous object recognition in the Göttingen minipig. Neural Plast..

[13] Kornum B, Knudsen G (2011). Cognitive testing of pigs (*Sus scrofa*) in translational biobehavioral research. Neurosci. Biobehav. Rev..

[14] Noldus LPJJ, Spink AJ, Tegelenbosch RAJ (2001). EthoVision: A versatile video tracking system for automation of behavioral experiments. Behav. Res. Methods Instrum. Comput..

[15] Ennaceur A, Delacour J (1988). A new one-trial test for neurobiological studies of memory in rats. 1: Behavioral data. Behav. Brain Res..

[16] Friess SH (2007). Neurobehavioral functional deficits following closed head injury in the neonatal pig. Exp. Neurol..

[17] Kozich JJ, Westcott SL, Baxter NT, Highlander SK, Schloss PD (2013). Development of a dual-index sequencing strategy and curation pipeline for analyzing amplicon sequence data on the MiSeq Illumina sequencing platform. Appl. Environ. Microbiol..

[18] Pavoine S, Ricotta C (2019). A simple translationfrom indices of speciesdiversity to indices of phylogenetic diversity. Ecol. Ind..

[19] Yue JC, Clayton MK (2005). A similarity measure based on species proportions. Commun. Stat. Theory Methods.

[20] *R: A Language and Environment for Statistical Computing* (R Foundation for Statistical Computing, 2015).

[21] Jeffery I, O'Toole P (2013). Diet–microbiota interactions and their implications for healthy living. Nutrients.

[22] Dinan TG, Cryan JF (2012). Regulation of the stress response by the gut microbiota: Implications for psychoneuroendocrinology. Psychoneuroendocrinology.

[23] Liew, W. P. P. *et al.* In *Beneficial Microorganisms in Medical and Health Applications* vol. 28 *Microbiology Monographs* (ed. Liong, M. T.) 223–255 (Springer, 2015).

[24] Pirbaglou M (2016). Probiotic supplementation can positively affect anxiety and depressive symptoms: A systematic review of randomized controlled trials. Nutr. Res..

[25] Farmer AD, Randall HA, Aziz Q (2014). It's a gut feeling: How the gut microbiota affects the state of mind. J. Physiol. Lond..

[26] Cryan, J. F. & Clarke, G. *International Review of Neurobiology—Gut Microbiome and Behavior*. vol. 131 (London: Academic Press, 2016). 10.1016/S0074-7742(16)30165-9 27793229

[27] Parashar A, Udayabanu M (2016). Gut microbiota regulates key modulators of social behavior. Eur. Neuropsychopharmacol..

[28] Lieberman H (2003). Nutrition, brain function and cognitive performance. Appetite.

[29] Gareau, M. G. In *Microbial Endocrinology: The Microbiota-Gut-Brain Axis in Health and Disease* vol. 817 *Advances in Experimental Medicine and Biology* (eds. Lyte, M. & Cryan, J. F.) 357–371 (Springer, 2014). 10.1007/978-1-4939-0897-4_1 24997027

[30] Wang B (2007). Dietary sialic acid supplementation improves learning and memory in piglets. Am. J. Clin. Nutr..

[31] Haagensen AMJ, Klein AB, Ettrup A, Matthews LR, Sorensen DB (2013). Cognitive performance of Gottingen minipigs is affected by diet in a spatial hole-board discrimination test. PLoS One.

[32] Liu HN (2014). Early supplementation of phospholipids and gangliosides affects brain and cognitive development in neonatal piglets. J. Nutr..

[33] Clouard C (2016). Prenatal, but not early postnatal, exposure to a Western diet improves spatial memory of pigs later in life and is paired with changes in maternal prepartum blood lipid levels. FASEB J..

[34] Sweis BM, Veverka KK, Dhillon ES, Urban JH, Lucas LR (2013). Individual differences in the effects of chronic stress on memory: Behavioral and neurochemical correlates of resiliency. Neuroscience.

[35] Bailey MT (2010). Stressor exposure disrupts commensal microbial populations in the intestines and leads to increased colonization by *Citrobacter rodentium*. Infect. Immun..

[36] Soderholm JD (2002). Chronic stress induces mast cell-dependent bacterial adherence and initiates mucosal inflammation in rat intestine. Gastroenterology.

[37] Cameron HL, Perdue MH (2005). Stress impairs murine intestinal barrier function: improvement by glucagon-like peptide-2. J. Pharmacol. Exp. Ther..

[38] Sun Y (2013). Stress-induced corticotropin-releasing hormone-mediated NLRP6 inflammasome inhibition and transmissible enteritis in mice. Gastroenterology.

[39] Peeters E (2005). Influence of supplemental magnesium, tryptophan, vitamin C, and vitamin E on stress responses of pigs to vibration. J. Anim. Sci..

[40] Zhang T (2015). Effects of dietary oregano essential oil supplementation on the stress response, antioxidative capacity, and HSPs mRNA expression of transported pigs. Livest. Sci..

[41] O'Driscoll K, Teixeira DL, O'Gorman D, Taylor S, Boyle LA (2013). The influence of a magnesium rich marine supplement on behaviour, salivary cortisol levels, and skin lesions in growing pigs exposed to acute stressors. Appl. Anim. Behav. Sci..

[42] Koopmans SJ (2005). Surplus dietary tryptophan reduces plasma cortisol and noradrenaline concentrations and enhances recovery after social stress in pigs. Physiol. Behav..

[43] Koopmans SJ (2006). Effects of supplemental L-tryptophan on serotonin, cortisol, intestinal integrity, and behavior in weanling piglets. J. Anim. Sci..

[44] Li YZ, Kerr BJ, Kidd KT, Gonyou HW (2006). Use of supplementary tryptophan to modify the behavior of pigs. J. Anim. Sci..

[45] Koopmans SJ, Ruis M, Dekker R, Korte M (2009). Surplus dietary tryptophan inhibits stress hormone kinetics and induces insulin resistance in pigs. Physiol. Behav..

[46] Zou Y (2017). Effects of dietary oregano essential oil and vitamin E supplementation on meat quality, stress response and intestinal morphology in pigs following transport stress. J. Vet. Med. Sci..

[47] Li JL (2013). Effects of dietary supplementation of chitosan on stress hormones and antioxidative enzymes in weaned piglets. J. Anim. Vet. Adv..

[48] Haagensen AMJ (2014). High fat, low carbohydrate diet limit fear and aggression in Gottingen minipigs. PLoS One..

[49] Clouard C, Gerrits WJJ, van Kerkhof I, Smink W, Bolhuis JE (2015). Dietary linoleic and a-linolenic acids affect anxiety-related responses and exploratory activity in growing pigs. J. Nutr..

[50] Ya BL (2013). Dietary cholesterol alters memory and synaptic structural plasticity in young rat brain. Neurol. Sci..

[51] Wang DS, Zheng W (2015). Dietary cholesterol concentration affects synaptic plasticity and dendrite spine morphology of rabbit hippocampal neurons. Brain Res..

[52] Ando S (1983). Gangliosides in the nervous system. Neurochem. Int..

[53] Ryan JM, Rice GE, Mitchell MD (2013). The role of gangliosides in brain development and the potential benefits of perinatal supplementation. Nutr. Res..

[54] Parois S, Calandreau L, Kraimi N, Gabriel I, Leterrier C (2017). The influence of a probiotic supplementation on memory in quail suggests a role of gut microbiota on cognitive abilities in birds. Behav. Brain Res..

[55] Lai MC, Huang LT (2011). Effects of early life stress on neuroendocrine and neurobehavior: mechanisms and implications. Pediatr. Neonatol..

[56] Backhed F (2015). Dynamics and stabilization of the human gut microbiome during the first year of life. Cell Host Microbe.

[57] Heijtz RD (2011). Normal gut microbiota modulates brain development and behavior. Proc. Natl. Acad. Sci..

[58] Wang X (2002). Evidences for vagus nerve in maintenance of immune balance and transmission of immune information from gut to brain in STM-infected rats. World J. Gastroenterol..

[59] Macpherson AJ, Harris NL (2004). Interactions between commensal intestinal bacteria and the immune system. Nature.

[60] Maynard CL, Elson CO, Hatton RD, Weaver CT (2012). Reciprocal interactions of the intestinal microbiota and immune system. Nature.

[61] Mizuno K, Giese KP (2005). Hippocampus-dependent memory formation: Do memory type-specific mechanisms exist?. J. Pharmacol. Sci..

[62] Cowansage KK, LeDoux JE, Monfils MH (2010). Brain-derived neurotrophic factor: A dynamic gatekeeper of neural plasticity. Curr. Mol. Pharmacol..

[63] Gareau M (2011). Bacterial infection causes stress-induced memory dysfunction in mice. Gut.

[64] Henry Y, Seve B, Mounier A, Ganier P (1996). Growth performance and brain neurotransmitters in pigs as affected by tryptophan, protein, and sex. J. Anim. Sci..

[65] McAfoose J, Baune BT (2009). Evidence for a cytokine model of cognitive function. Neurosci. Biobehav. Rev..

[66] Jenkins TA, Nguyen JCD, Polglaze KE, Bertrand PP (2016). Influence of tryptophan and serotonin on mood and cognition with a possible role of the gut-brain axis. Nutrients.

[67] Matsumoto M (2013). Cerebral low-molecular metabolites influenced by intestinal microbiota: A pilot study. Front. Syst. Neurosci..

[68] Schmitt JAJ, Wingen M, Ramaekers JG, Evers EAT, Riedel WJ (2006). Serotonin and human cognitive performance. Curr. Pharm. Des..

[69] Yan W (2019). Efficacy of fecal sampling as a gut proxy in the study of chicken gut microbiota. Front. Microbiol..

[70] O’brien MER (2004). SRL172 (killed *Mycobacterium vaccae*) in addition to standard chemotherapy improves quality of life without affecting survival, in patients with advanced non-small-cell lung cancer: Phase III results. Ann. Oncol..

[71] Lowry CA (2007). Identification of an immune-responsive mesolimbocortical serotonergic system: potential role in regulation of emotional behavior. Neuroscience.

[72] Matthews DM, Jenks SM (2013). Ingestion of *Mycobacterium vaccae* decreases anxiety-related behavior and improves learning in mice. Behav. Process..

[73] Nhung BT (2009). Impact of milk consumption on performance and health of primary school children in rural Vietnam. Asia Pac. J. Clin. Nutr..

[74] Van Der Linden M, Meulemans T, Marczewski P, Collette F (2000). The relationships between episodic memory, working memory, and executive functions: The contribution of the prefrontal cortex. Psychol. Belgica.

