# Supplementary material for: Potential improvements of the cognition of piglets through a synbiotic supplementation from 1 to 28 days via the gut microbiota
Source: Sci Rep. 2021 Dec 16;11:24113. doi: 10.1038/s41598-021-03565-5 (PMC8677727; doi:10.1038/s41598-021-03565-5)

Supplementary Figure: Schematic of the device used for the T-maze test. For each trial, one of the North or South arm is randomly closed. The North and the South arms are the arms to start the test, whereas the West and East arms are the arms containing the bowls (1 with a reward and 1 without). Each arm has a different visual pattern.


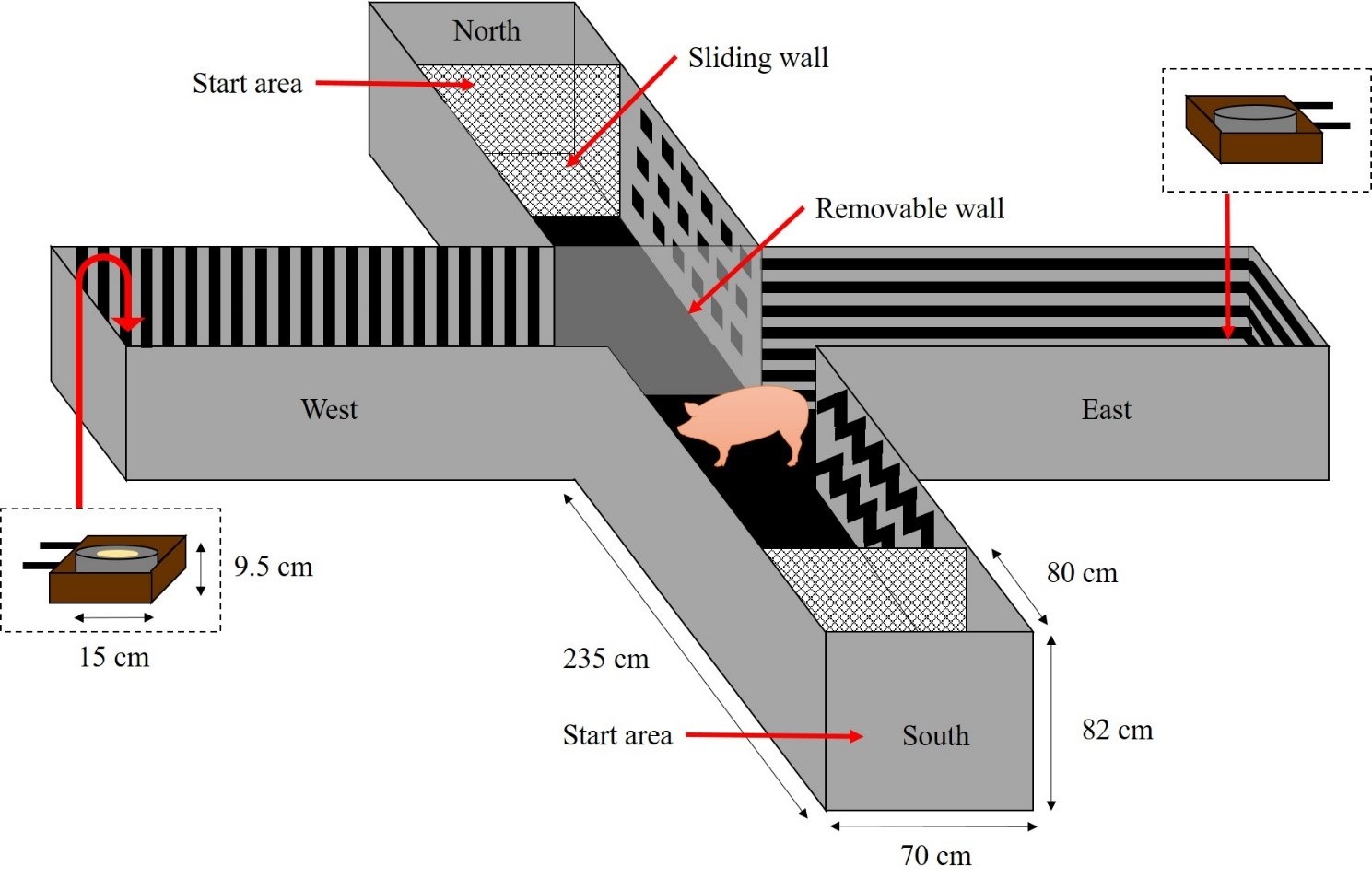

Supplement: Supplementary file 1 — Supplementary Figures. [file 41598_2021_3565_MOESM1_ESM.docx]
