# Supplementary material for: Potential improvements of the cognition of piglets through a synbiotic supplementation from 1 to 28 days via the gut microbiota
Source: Sci Rep. 2021 Dec 16;11:24113. doi: 10.1038/s41598-021-03565-5 (PMC8677727; doi:10.1038/s41598-021-03565-5)
Supplement: Supplementary file 2 — Supplementary Table 1. [file 41598_2021_3565_MOESM2_ESM.docx]

Supplementary Table 1: Pearson correlations between cognitive traits durations from three different cognitive tests (SOR: Spontaneous Object Recognition test; BARR: Fence barrier task; TMAZE: Spatial T-maze task).^1^

| Traits^2^ |  | **SOR** | | | **BARR** | | | | | **TMAZE** | | | | | | | | |
| --- | --- | --- | --- | --- | --- | --- | --- | --- | --- | --- | --- | --- | --- | --- | --- | --- | --- | --- |
|  |  | Inter_NewObject_Dur (s) | Inter_OldObject_Dur (s) | Discriminant Index | Trial1_Duration (s) | Trial2_Duration (s) | Trial3_Duration (s) | Trial4_Duration (s) | Trial5_Duration (s) | A3_MeanDuration (s) | A4_MeanDuration (s) | A5_MeanDuration (s) | A6_MeanDuration (s) | R1_MeanDuration (s) | R2_MeanDuration (s) | R3_MeanDuration (s) | Reversal_1stSuccess (n) | Reversal_2Success (n) |
| **SOR** | Inter_NewObject_Dur (s) |  | **0.36** | -0.04 | 0.12 | 0.05 | **0.43** | -0.11 | 0.04 | **0.55** | *0.38* | 0.22 | 0.29 | 0.23 | -0.19 | 0.23 | -0.04 | 0.15 |
|  | Inter_OldObject_Dur (s) |  |  | -0.02 | *0.32* | 0.19 | -0.14 | -0.26 | -0.13 | -0.02 | 0.02 | 0.22 | 0.08 | 0.33 | 0.06 | **0.52** | 0.13 | **0.61** |
|  | Discriminant Index |  |  |  | -0.09 | -0.15 | 0.29 | 0.20 | 0.22 | 0.16 | 0.23 | 0.14 | 0.29 | -0.24 | 0.04 | -0.18 | -0.14 | -0.18 |
| **BARR** | Trial1_Duration (s) |  |  |  |  | 0.27 | -0.05 | -0.05 | -0.19 | -0.07 | -0.06 | 0.06 | -0.04 | -0.10 | -0.18 | 0.17 | **0.61** | **0.56** |
|  | Trial2_Duration (s) |  |  |  |  |  | 0.09 | *0.37* | 0.10 | -0.31 | -0.06 | -0.05 | -0.18 | -0.49 | 0.08 | 0.37 | -0.15 | 0.001 |
|  | Trial3_Duration (s) |  |  |  |  |  |  | **0.67** | **0.51** | **0.76** | -0.01 | 0.002 | -0.07 | 0.07 | -0.12 | -0.03 | -0.25 | -0.09 |
|  | Trial4_Duration (s) |  |  |  |  |  |  |  | **0.69** | **0.43** | -0.01 | *0.40* | 0.08 | 0.04 | -0.27 | -0.09 | -0.14 | -0.33 |
|  | Trial5_Duration (s) |  |  |  |  |  |  |  |  | 0.17 | -0.04 | 0.02 | -0.05 | -0.24 | 0.39 | 0.08 | -0.19 | -0.21 |
| **TMAZE** | A3_MeanDuration (s) |  |  |  |  |  |  |  |  |  | **0.50** | 0.34 | 0.33 | 0.36 | -0.09 | 0.10 | 0.20 | 0.15 |
|  | A4_MeanDuration (s) |  |  |  |  |  |  |  |  |  |  | **0.65** | *0.35* | 0.21 | 0.26 | **0.62** | -0.22 | -0.01 |
|  | A5_MeanDuration (s) |  |  |  |  |  |  |  |  |  |  |  | **0.53** | 0.35 | 0.25 | **0.53** | -0.09 | 0.26 |
|  | A6_MeanDuration (s) |  |  |  |  |  |  |  |  |  |  |  |  | 0.03 | -0.02 | 0.31 | 0.29 | 0.17 |
|  | R1_MeanDuration (s) |  |  |  |  |  |  |  |  |  |  |  |  |  | 0.08 | *0.53* | -0.34 | 0.23 |
|  | R2_MeanDuration (s) |  |  |  |  |  |  |  |  |  |  |  |  |  |  | 0.40 | 0.20 | 0.35 |
|  | R3_MeanDuration (s) |  |  |  |  |  |  |  |  |  |  |  |  |  |  |  | -0.08 | 0.16 |
|  | Reversal_1stSuccess (n) |  |  |  |  |  |  |  |  |  |  |  |  |  |  |  |  | **0.69** |
|  | Reversal_2Success (n) |  |  |  |  |  |  |  |  |  |  |  |  |  |  |  |  |  |

^1^Within the table values in **bold** have a *P*-value < 0.05 and in *italic* have a *P*-value < 0.1.

^2^Inter_NewObject_Dur: total duration of interaction with the new object; Inter_OldObject_Dur: total duration of interaction with the old object; _MeanDuration: average duration measured for the 10 successive trials of each daily session; A3 to A6: days 3 to 6 during the acquisition stage of the test; R1 to R3: days 1 to 3 during the reversal stage of the test; Reversal_1stSuccess: the number of trials needed to succeed for the first time in the reversal stage; Reversal_2Success: the number of trials needed to do two successive trials in the reversal stage; Discriminant Index: (DN1: Duration interacting with the novel object – DO3: Duration interaction with the old object)/(DN1 + DO3).
