# Supplementary material for: Potential improvements of the cognition of piglets through a synbiotic supplementation from 1 to 28 days via the gut microbiota
Source: Sci Rep. 2021 Dec 16;11:24113. doi: 10.1038/s41598-021-03565-5 (PMC8677727; doi:10.1038/s41598-021-03565-5)
Supplement: Supplementary file 3 — Supplementary Table 2. [file 41598_2021_3565_MOESM3_ESM.docx]

Supplementary Table 2: Significant Pearson correlations between cognitive traits from three different cognitive tests (SOR: Spontaneous Object Recognition test; BARR: Fence barrier task; TMAZE: Spatial T-maze task) and bacterial taxa.^1^

| Test | Traits^2^ | Pvalue | R | Classified OTUs statistically associated with behavioral traits *(Genus level)*^3^ |
| --- | --- | --- | --- | --- |
| SOR | Total duration of interaction with the new object (s) | NS |  |  |
|  | Total duration of interaction with the old object (s) | NS |  |  |
| BARR | Trial 1 duration (s) | NS |  |  |
|  | Trial 2 duration (s) | 0.05 | -0.32 | *Prevotella* |
|  |  | 0.05 | 0.31 | *Clostridium* XVIII |
|  |  | 0.05 | 0.31 | *Faecalicoccus* |
|  |  | 0.05 | 0.35 | *Clostridium, XlVa* |
|  |  | 0.05 | 0.36 | *Ruminococcaceae* unclassified |
|  | Trial 3 duration (s) | NS |  |  |
|  | Trial 4 duration (s) | NS |  |  |
|  | Trial 5 duration (s) | NS |  |  |
| TMAZE | Mean duration to touch the rewarded bowl_A3 (s) | NS |  |  |
|  | Mean duration to touch the rewarded bowl_A4 (s) | NS |  |  |
|  | Mean duration to touch the rewarded bowl_A5 (s) | NS |  |  |
|  | Mean duration to touch the rewarded bowl_A6 (s) | NS |  |  |
|  | Mean duration to touch the rewarded bowl_R1 (s) | NS |  |  |
|  | Mean duration to touch the rewarded bowl_R2 (s) | NS |  |  |
|  | Mean duration to touch the rewarded bowl_R3 (s) | NS |  |  |
|  | First time to try the new rewarded arm (n) | NS |  |  |
|  | First two successive trials during the reversal stage (n) | NS |  |  |

^1^Significant OTU associations with behavioral traits were analyzed using the commands “corr.axes" and “otu.association” in mothur from the mothur standard operating procedure (SOP) designed for MiSeq data^75^. The mothur MiSeq SOP was accessed in August 2018.

^2^A3 to A6: days 3 to 6 during the acquisition stage of the test; R1 to R3: days 1 to 3 during the reversal stage of the test.

^3^Bacteria mentioned had a p-value below 0.001.
